# “Friends or foes”: a new perspective of tumour metabolic transcriptional modification

**DOI:** 10.1038/s41419-025-07429-y

**Published:** 2025-02-17

**Authors:** Tong Shi, Qishun Geng, Zhaoran Wang, Chaoying Wen, Jiahe Xu, Yi Jiao, Wenya Diao, Jienan Gu, Tingting Deng, Cheng Xiao, Baoyuan Zhong, Jianfeng Wang

**Affiliations:** 1 China-Japan Friendship Clinical Medical College, Chinese Academy of Medical Sciences & Peking Union Medical College, Beijing, China; 2 Institute of Clinical Medical Sciences, China-Japan Friendship Hospital, Beijing, China; 3 Peking University China-Japan Friendship School of Clinical Medicine, Beijing, China; 4 Beijing University of Chinese Medicine, China-Japan Friendship Hospital Clinical Medicine, Beijing, China; 5 Department of Emergency, China-Japan Friendship Hospital, Beijing, China; 6 Department of General Surgery, First Affiliated Hospital of Gannan Medical University, Ganzhou, China; 7 Department of Urology, China-Japan Friendship Hospital, Beijing, China

**Keywords:** Cancer metabolism, Cancer epigenetics

## Abstract

Energy metabolism plays a pivotal role in cancer clinical treatment and has become an important means of clinical diagnosis of tumour progression. However, current research mostly focuses on changes in metabolic products and neglects the deeper mechanisms of transcriptional regulation. This paper proposes a new perspective, establishing a comprehensive network that reveals the interaction between metabolism and transcription, which explores how tumour metabolism affects tumour progression through transcriptional modifications, and provides a novel approach for optimizing tumour treatment strategies. This viewpoint is conducive to overcoming current bottlenecks in treatment and promoting the development of drug combinations and personalized medicine.

## Highlights

- Metabolic transcriptional factors form a complex interactive network in tumour.
- There is heterogeneity in tumour metabolic transcriptional function.
- Transcriptional regulation can act synergistically or antagonistically, rather than independently.

## Introduction

The continuous proliferation of tumour cells requires a constant supply of energy [1]. Studies have shown that normal human cells mainly generate the energy they need through aerobic respiration, namely, oxidative phosphorylation (OXPHOS) [2], whereas tumour cells are more inclined to use aerobic glycolysis to generate the adenosine triphosphate (ATP) that is necessary for their growth and metastasis [3]. Tumour glycolytic pathways can be regulated by epigenetic modifications like pre-transcriptional and post-transcriptional regulation, such as m^6^A methylation and involvement of transcription factors [4]. This article reviews recent research progress on understanding the roles and crosstalk of transcription factors in regulating tumour cell glycolysis to identify new targets and strategies for the clinical treatment of tumours [5].

Transcription factors are a class of proteins that bind to gene promoter sequences to regulate gene transcription or alter gene expression. In addition to traditional MYC family members, some transcription factors, such as SALL4, ATOH8, HOXA9, and MZF1, can directly or indirectly regulate the expression of glycolysis-related genes [5]. Abnormal activation of the PI3K/Akt signalling pathway is the main mechanism underlying tumour glucose metabolism. In recent years, several new signalling pathways, such as the YY1/MZF1, VEGFR2/AKT/ATOH8, and CD36/GPC4/β-catenin/MYC pathways, have been shown to be involved in the regulation of glycolysis. In addition, studies on the subcellular localization and functional diversity of key enzymes that are involved in glycolysis have attracted increasing attention.

## Characterization of the glycolytic pathway in tumour cells

**Fig. 1:**
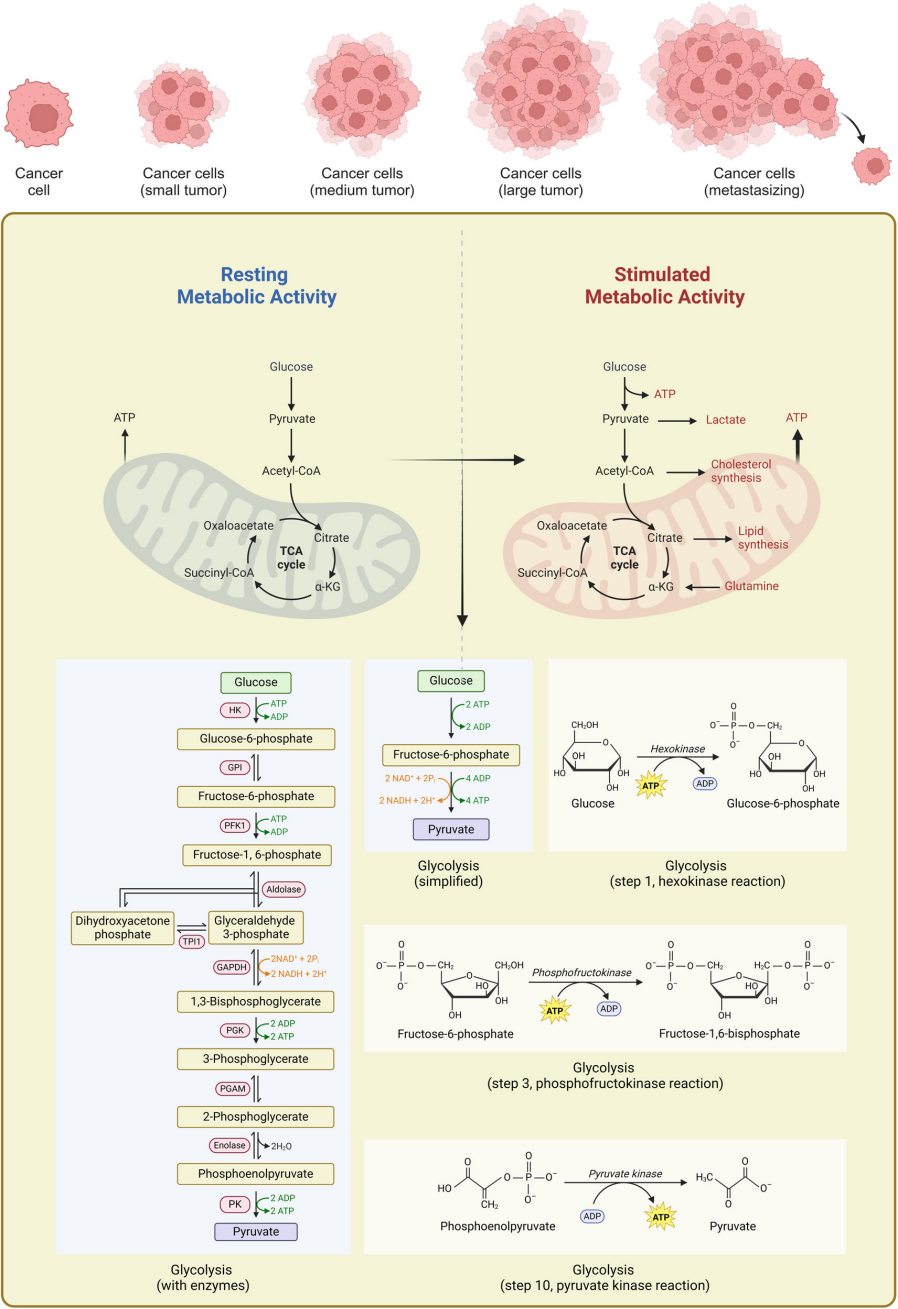
Overview of the glycolytic metabolic pathway in tumour growth. Tumour tissue is often accompanied by abnormal blood vessel growth to supply more energy. Free glucose is taken up by cells from the tumour microenvironment and is ultimately converted into lactate through the catalysis of key enzymes such as HK2, PFK1, and PK, causing the further acidification of the tumour microenvironment. Abnormal blood vessel growth leads to hyperactivation of glycolytic function in tumours, further producing more ATP and metabolic byproducts that promote angiogenesis. This figure is created with BioRender.com.

Aerobic glycolysis is an abnormal bioenergetic activity [4]. Even in the presence of sufficient oxygen levels, tumour cells can utilize large amounts of glucose for fermentation to produce lactic acid via glycolysis while maintaining a low OXPHOS rate. The glycolytic pathway is a relatively complex biochemical reaction chain that occurs mainly in the cytoplasm, and most of the reaction processes within this pathway are reversible. However, the three steps that are catalyzed by hexokinase (HK), phosphofructokinase (PFK), and pyruvate kinase (PKM), which are the rate-limiting steps of the glycolytic pathway, are irreversible. The activities of key enzymes in the glycolytic pathway determine the rate of ATP production, and many glycolysis-related transcription factors affect tumour cell glycolysis by targeting these key enzymes (Fig. 1).

## Regulation of glycolysis by transcription factors in tumour cells

In contrast to normal cells, tumour cells are persistently in a hypoxic state [6]. Under hypoxic stress conditions, certain transcription factors undergo mutation, deletion, or amplification, which directly or indirectly modulates the expression levels of key glycolysis-related enzymes in tumour cells, thereby governing the rate of glycolysis [7]. Among these transcription factors, a growing body of studies has demonstrated that several classical transcription factors, such as members of the MYC family, HIF-1α, NF-κB, and members of the STAT family, assume crucial roles in the glycolysis pathway. In recent years, additional transcription factors, including SALL4, SMAD4, HOXA9, ATOH8, and MZF1, have been revealed to execute novel functions in the regulation of glycolysis. Moreover, intricate interactions transpire among diverse transcription factors, and the corresponding research outcomes are still being updated (Table 1).

**Table 1:** Transcription factors that regulate tumour cell glycolysis.

Transcription factor	Regulator targets	Tumour types	Function	References
ATOH8	HK2	CTC	Promote	[20]
BACH1	HK2/GAPDH	Lung cancer	Promote	[24]
CBX4	ALDOB	Melanoma etc.	Inhibit	[73]
c-Jun	GLUT1	Breast cancer	Inhibit	[74, 75]
E2F1	PDK/PFKFB	Retinoblastoma	Promote	[76, 77]
EGR1	PFKL	HCC	Inhibit	[78]
FOX	HK2/GLUT1/LDH	Colorectal/Liver/Pancreatic cancer	Promote/ Inhibit	[79–81]
HIF-1α	HK2/LDH/PDK/GLUT1	Burkitt’s lymphoma/HCC *etc*.	Promote	[82–84]
HOXA9	HK2/GLUT1/PDK	cSCC	Inhibit	[40]
IRF7	PKM2	Osteosarcoma	Inhibit	[85]
KDM6A	HK2/PKM2	Glioblastoma	Promote	[86]
KLF14	HK2/LDHA	HCC/Colorectal cancer	Promote/Inhibit	[48, 49]
LHX9	PKM2	Gastric cancer/Ovarian cancer	Promote	[87, 88]
MYC	HK2/LDH/PKM/PGAM	Nasopharyngeal/Carcinoma/Lymphoma/HNC/OS *etc*.	Promote	[89–91]
MZF1	HK2/PGK	NB	Promote	[23]
NFAT5	GLUT1/PGK1	Pancreatic cancer/ICC	Promote	[92, 93]
NF-κB	PKM	Brain tumour *etc*.	Promote	[11]
OVOL2	GLUT1/HK2/ALDOA/PGAM1/PKM2/LDHA	Breast Cancer	Inhibit	[94]
p53	HK2/LDHA/PGAM	Breast cancer/Cervical cancer	Inhibit	[12, 45, 95, 96]
SALL4	GLUT1/HK2	HCC/Gastric cancer	Promote	[18, 97–99]
SIRT	GLUT1/LDHA	Colorectal carcinoma/Prostate cancer/HCC	Promote	[100–102]
SIX1	HK2/PKM/PGK/LDH/PFKL	Breast cancer	Promote	[103]
SMAD4	PFKFB/HK2/PGK	Glioblastoma	Inhibit	[21, 22, 39]
STAT3	HK2/PKM	Ovarian cancer/Liver precancerous lesions/Breast cancer/HCC	Promote	[14, 15, 104–107]
TEAD	HK2	Breast cancer	Promote	[58]
ZEB1	GLUT3/HK2/PFKP/PFKM/PKM2	HCC/NEPC/Breast cancer/OSCC/Ovarian cancer	Promote	[32, 33, 108–110]

*ATOH8* atonal BHLH transcription factor 8, *MZF1* myeloid zinc finger 1, *BACH1* BTB domain and CNC homology 1, *CBX4* chromobox 4, *c-Jun* transcription factor AP-1-like, *MYC* transcriptional regulator MYC-like, *E2F1* E2F transcription Factor 1, *SALL4* spalt like transcription factor 4, *SMAD4* small mothers against decapentaplegic family member 4, *HOXA9* homeobox A9, *EGR1* early growth response 1, *FOX* forkhead box, *HIF-1α* hypoxia inducible factor 1 subunit alpha, IRF7 interferon regulatory factor 7, *KDM6A* lysine (K)-specific demethylase 6A, *KLF14* Kruppel-like transcription factor 14, *LHX9* LIM homeobox protein 9, *NFAT5* nuclear factor of activated T cells 5, *NF-κB* nuclear factor kappa B, *OVOL2* Ovo like zinc finger 2, TP53 tumour protein p53, *SIRT* sirtuin, *SIX1* sine oculis-related homeobox 1, *STAT3* signal transducer and activator of transcription 3, TEAD TEA domain transcription factor, *ZEB1* zinc finger E-box binding homeobox 1, *CTC* circulating tumour cell, *NB* neuroblastoma, *HNC* head and neck cancer, *OS* osteosarcoma, *HCC* hepatocellular carcinoma, *PDAC* pancreatic ductal adenocarcinoma, *cSCC* squamous cell carcinomas, *CTC* circulating tumour cell, *ICC* intrahepatic cholangiocarcinoma, *GAPDH* glyceraldehyde-3-phosphate dehydrogenase, *HK* hexokinase, *LDH* lactate dehydrogenase, *GLUT* glucose transporter, PGK phosphoglycerate Kinase, *PKM* pyruvate Kinase M1/2, *PGAM* phosphoglycerate mutase, *PDK* pyruvate dehydrogenase kinase, ALDOA/B aldolase, fructose-bisphosphate A/B, *PFKFB* 6-phosphofructo-2-kinase/fructose-2,6-bisphosphatase, *PFKP* phosphofructokinase, platelet, *PFKL* phosphofructokinase, liver, *LDH* lactate dehydrogenase.

### Mechanisms by which transcription factors regulate glycolysis in tumour cells

**Fig. 2:**
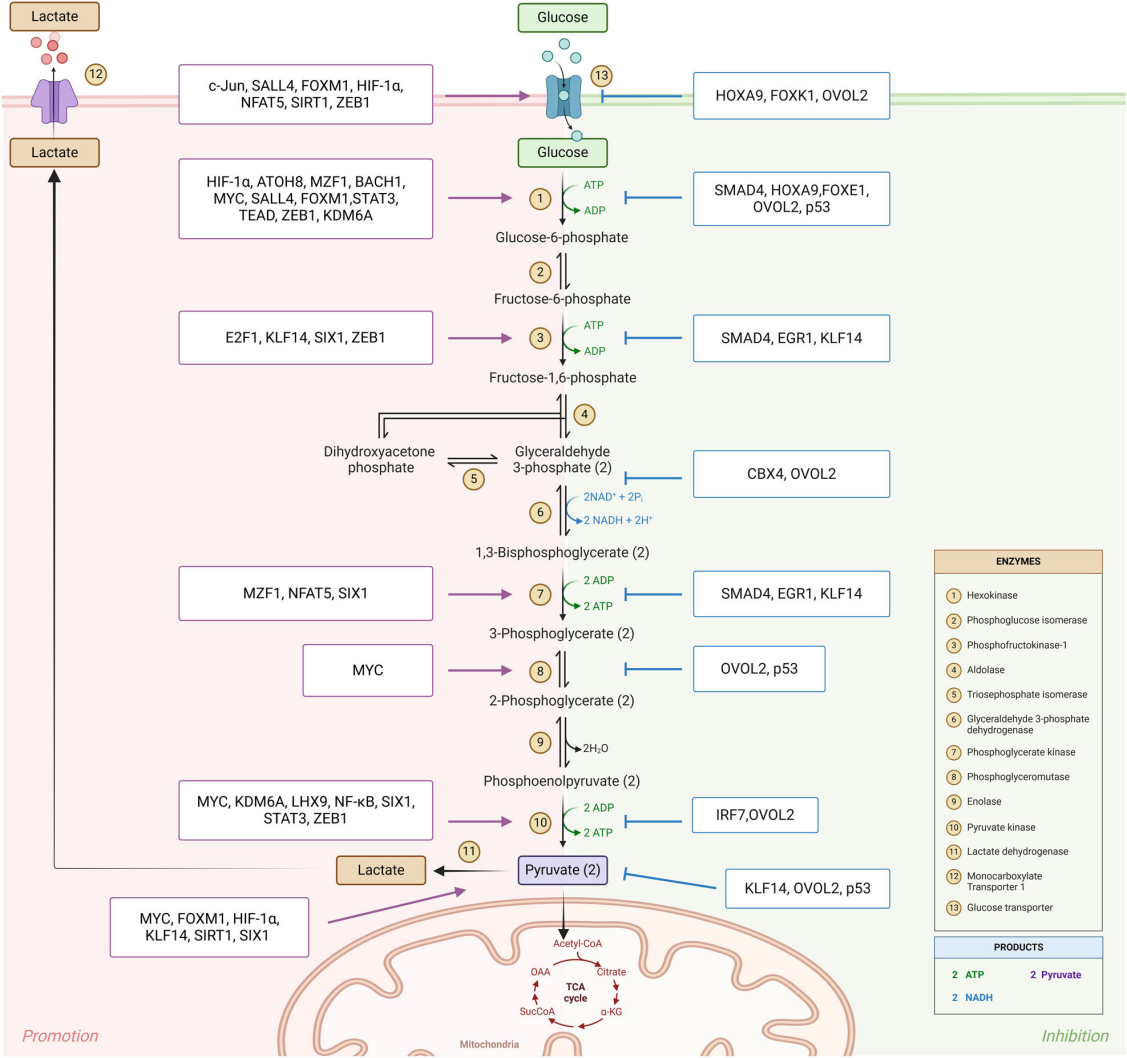
Cooperative and inhibitory networks of transcriptional regulation in tumour glycolysis. The regulatory effects of transcription factors on glycolysis in tumour cells are very complex. One transcription factor can regulate the expression of multiple target genes, and the functions of different transcription factors synergistically promote or antagonize each other. In addition, the targets of classical transcription factors (such as HIF-1α, MYC, p53, E2F1, and p53) are complex and often involve multiple key enzymes. However, some recently discovered transcription factors (such as SALL4, ATOH8, SMAD4, and MZF1) have relatively few, even single, targets, and the specific mechanism remains to be elucidated. This figure is created with BioRender.com.

The regulatory mechanism through which transcription factors impact glycolysis in tumour cells constitutes a complex network. On the one hand, a plurality of transcription factors is capable of interacting to assemble a transcription initiation complex and engage in the transcription of a single gene. On the other hand, identical transcription factors can also attach to the promoter regions of distinct genes, thereby influencing glycolysis in tumours through multiple targets (Fig. 2).

#### Transcription factors that promote glycolysis

##### HIF-1

HIF-1 is a heterodimeric protein that consists of two subunits, namely, the hypoxia-inducible heterodimer HIF-1α and the constitutive heterodimer HIF-1β, that bind to the promoter regions of specific nuclear genes and regulate their transcription. Typically, HIF-1 is highly expressed in tumour cells, and high levels of HIF-1 protein expression can usually be detected in metastatic tumours; however, HIF-1 is expressed at relatively low levels in benign tumours and nontumour cells [8]. Early studies demonstrated that HIF-1 activates vascular endothelial growth factor (VEGF) transcription and is involved in hypoxia-mediated apoptosis, proliferation and angiogenesis [9, 10]. Moreover, HIF-1α has been shown to play an important role in tumour energy metabolism. Most of the genes that encode glycolysis-related enzymes and transporter proteins (Fig. 2) are downstream targets of HIF-1α, except for the genes that encode glycosylphosphatidylinositol (GPI) and monocarboxylate transporter (MCT) [5]. Therefore, HIF-1α is considered as one of the most critical transcription factors in the glycolytic pathway in tumour cells.

##### NF-κB

NF-κB is a nuclear transcription factor that is present in almost all animal cells, and it is involved in the transcriptional regulation of multiple metabolic processes that are associated with tumour progression. The structures of the NF-κB subunits p50, p52, RelA, RelB and c-Rel all contain recognition sites for the promoters of target genes. When NF-κB is transcriptionally active, it requires only two subunits to form functional homodimers or heterodimers. RelA subunits are mutated in breast, lung and ovarian cancers. Compared with normal cells, RelA-overexpressing cells exhibit accelerated extracellular glucose consumption and significantly increased intracellular lactate and ATP production. In addition, hypoxia activates NF-κB by regulating the activation of IkB and IκBα kinase (also known as IKK), which directly upregulates PKM2 expression at the transcription level, thus promoting tumour cell glycolysis [11, 12]. Thus, NF-κB also plays a key role in promoting metabolic reprogramming in tumour cells.

##### STAT family

The signal transducer and activator of transcription (STAT) family includes transcription factors that are involved in a variety of biological processes. The STAT family includes seven subfamilies (STAT1-4, STAT-5a, STAT-5b and STAT-6); among these subfamilies, STAT3 and STAT5 are the most highly expressed and activated in malignant tumours and have received widespread attention. There are two isoforms of STAT3 (α and β), and STAT3α is relatively well studied in the context of cancer. Upon activation by cytokines, growth factors and JAKs (Janus kinases), phosphorylated STAT3 (pSTAT3) dimerises and binds to the promoters of its target genes to activate their transcription. Recent studies have confirmed that STAT3 can directly or indirectly regulate tumour cell glycolysis. In hepatocellular carcinoma, STAT3 increases the expression of GLUT1 in HBV- and HCV-infected cancer cells and increases glucose consumption and lactate production in HepG2 and Hep3B hepatocellular carcinoma cells [5]. In MCF-7 breast cancer cells, STAT3 also directly binds to the promoter region of PKM2 and increases its transcription [13]. Moreover, STAT3 can also indirectly promote the expression of PKM2 by upregulating the expression of heterogeneous cytosolic ribonucleoprotein A1 (hnRNP-A1) to enhance the glycolytic activity of breast cancer cells [14]. Interestingly, PKM2 can also act as a transcription factor to inversely activate STAT3, thereby promoting Th17 cell differentiation and autoimmune-mediated inflammatory responses [15]. However, the function of this interaction in glucose metabolism is not known.

##### Others

E2F1, which is the first-described and most well-studied member of the E2F family, is particularly important because of its function in cellular metabolism. E2F1 acts as a transcription factor that directly targets key enzymes of glycolysis to exert regulatory effects. For example, E2F1 directly binds to the promoter region of the PDK4 gene, thereby limiting glucose oxidation in mitochondria [16]. PFKFB, which is a key glycolytic enzyme that is involved in cell proliferation, is also a downstream target gene of E2F1 [17].

SALL4 is a C2H2 (Cys2His2) zinc finger transcription factor that acts as a transcriptional activator or repressor. The role of SALL4 as a transcriptional activator has also received substantial attention, especially in the context of energy metabolism. Recent studies have shown that SALL4 can directly bind to the promoter region of HK2 in gastric cancer cells and promote tumour cell glycolysis at the transcription level [18].

ATOH8, which is a basic helix-loop transcription factor, including the development of the cardiovascular, skeletal muscle and nervous systems, as well as the establishment of haematopoiesis. In previous studies, the expression of ATOH8 in tumours was shown to vary depending on the tumour type, and its tumour-promoting or tumour-inhibiting effects remain controversial [19]. A recent study revealed the role of ATOH8 in tumour cell glycolysis, demonstrating that ATOH8 transcriptionally activates HK2-mediated glycolysis, thereby increasing the survival of circulating tumour cells (CTCs) and completing the upstream regulatory network of HK2 [20].

SMAD4 plays a key role in tumourigenesis as a core mediator of the typical TGF-β signalling pathway. In glioblastoma, the SMAD4 transcription factor complex directly binds to the promoter region of PFKFB3 and increases tumour cell glycolysis by facilitating PFKFB3 transcription [21]. Studies in a mouse model of lung fibrosis revealed that HK2 may be involved in regulating cellular glycolytic processes as a downstream molecule of the TGF-β/SMAD signalling pathway [22]. These studies elucidated the mechanism by which the TGF-β/SMAD pathway regulates cellular energy metabolism.

MZF1 is a member of the Kruppel family of proteins and essential for the differentiation, proliferation and migration of haematopoietic cells, which can directly upregulate the expression of HK2 and PGK1, which are the key enzymes of glycolysis, and promote tumour cell glycolysis [23].

BACH1, within the BTB and CNC homology (BACH) family that contain a leucine zipper (bZIP) structural domain and a Bric-a-brac-Tramtrack-Broad complex (BTB) structural domain, is highly expressed in neutrophils, NK cells, monocytes, macrophages and dendritic cells [17]. In lung cancer, BACH1 directly targets the promoters of the key glycolytic enzymes HK2 and GAPDH, upregulate their mRNA and protein expression, further promote intracellular ATP synthesis, and induce the growth and metastasis of tumour tissues. In addition, antioxidants can increase the rate of glycolysis by increasing the protein stability of BACH1 in lung cancer cells [24].

SIX1 is a transcriptional activator or repressor involved in development and differentiation. It is essential for the expansion of progenitor cell populations and intercellular communication, especially in the early stages of development. SIX1 lacks an activation domain and requires a cofactor to function. Among its cofactors, SIX1 binds to EYA family proteins and mediates their nuclear translocation to activate transcription and acts synergistically with domain adaption using cross-domain homomorphism (DACH) family members to increase the expression of many glycolysis-related genes, such as GLUT1 and HK2, thus promoting tumour growth and inducing the Warburg effect [23].

Sirtuins belong to the third class of histone deacetylases, and their enzymatic activity relies on nicotinamide adenine dinucleotide (NAD+) [25]. SIRT1 extensively regulates metabolic processes via histone and nonhistone deacetylation. HIF1α is a direct target of SIRT1. Sometimes, SIRT1 and HIF1α cooperate or act separately to mediate immune responses. Additionally, SIRT1 can deacetylate several downstream targets, including NF-κB and TP53 [26, 27]. SIRT1 can also regulate immune responses directly through the deacetylation of some key transcription factors or indirectly through metabolic pathways [28–31].

ZEB1 is generally considered a transcription factor that directly activate or repress gene expression by binding to the regulatory regions of target genes [32] such as HK2, PFKP, PFKM and PKM2, which are glycolytic rate-determining enzymes to promote the Warburg effect, proliferation, migration, and chemoresistance of breast cancer [32, 33]. In addition, ZEB1 exerts its biological effects to increase glycolysis in response to hypoxic conditions via the PI3K/Akt/HIF-1α signalling axis, which contributes to fostering an immunosuppressive tumour microenvironment (TME) [32, 34–38].

#### Transcription factors that inhibit glycolysis

##### FOX protein family members

The FOX protein family has many members, ranging from FOXA to FOXS, and these members are divided into 19 subfamilies with very different modes of action. For example, the FOXA subfamily mediates cell differentiation and morphological maintenance; the FOXM subfamily regulates the cell cycle; and the FOXO subfamily controls cellular energy metabolism. The roles of these proteins in tumours are also diverse; for example, FOXC and FOXM subfamily members mostly act as oncogenic factors, whereas others, such as FOXF and FOXJ subfamily members, act as tumour suppressors. Among these subfamilies, the FOXO subfamily consists of the FOXO-1, FOXO-3a, FOXO-4 and FOXO-6 proteins. FOXO-1 downregulates the mRNA levels of enolase (ENO) and PKM in the mouse liver and acts as a downstream target of pAKT to block the transcription of glucose-6-phosphatase and phosphoenolpyruvate kinase in hepatocytes. FOXO-3a inhibits tumour cell glycolysis by transactivating the TSC1 molecule, which is downstream of mTORC1. In addition, in colorectal cancer, FOXE1 directly binds to the promoter region of HK2 and negatively regulates its transcription [39]. FOXK1 directly acts on the Akt/mTOR signalling pathway in hepatocellular carcinoma cells, inhibiting cell growth and glycolytic activity [40]. In addition to glycolysis inhibition, members of the FOX family promote glycolysis. For example, FOXO-6 activates the PI3K/Akt/mTOR pathway in colorectal cancer (CRC) cells and alters cellular metabolism by promoting glycolysis and inhibiting mitochondrial respiration, demonstrating the importance of the FOXO subfamily in the regulatory network of tumour cell glycolysis. In breast and ovarian cancers, FOXM1 promotes tumour growth and metastasis by increasing glucose uptake and lactate production in tumour cells via the upregulation of the protein levels of LDHA, GLUT1 and HK2 [20, 23]. In conclusion, although the FOX protein family comprises transcription factors that were discovered early and have been widely studied, its development is promising and still attracts the attention of many researchers.

##### HOXA9

HOX genes encode a series of transcription factors that are essential for controlling cell differentiation during embryonic development, and their expression in tumours, especially that of HOXA9, is closely associated with tumourigenesis [41]. Early studies confirmed that HOXA9 expression is closely associated with the abnormal proliferation of acute myeloid leukaemia (AML) cancer cells, and it is an important oncogene in haematological malignancies; however, its role in solid tumours is not singular. In recent years, it has been shown that the oncogenic effect of HOXA9 in cutaneous squamous cell carcinoma (cSCC) may be related to glycolysis. In cSCC, HOXA9 can interact with the transcription factor Cysteine-rich protein 2 (CRIP2) to limit glucose uptake and utilization by negatively regulating the expression of HIF-1α and its downstream glycolytic genes [40]. Moreover, in leukaemia cells, HOXA9 interacts with MYC genes, suggesting that HOXA9 regulates tumour cell glycolysis.

##### RB1

The retinoblastoma gene (Retinoblastomal, RB1) was the first tumour suppressor gene to be identified, and the gene is mutated at different frequencies in a variety of human cancers. Although RB1 itself is not a transcription factor, the association of E2F with RB family members represses target genes. When E2F is phosphorylated by the cyclin-dependent kinase (CDK) complex, RB is released, facilitating the promotion of downstream gene transcription by E2F. Evidence about the roles of RB family members in energy metabolism is also emerging. Recent studies have shown that RB1 deficiency promotes tumour cell glycolysis but does not affect the rate of OXPHOS in a mouse model of lung cancer [42]. In contrast, in breast cancer, RB1 deletion increases the expression of the mitochondria-associated protein molybdopterin (MPT) in tumour cells and increases the rate of OXPHOS [43]. Although no studies have shown a direct regulatory effect of RB on glycolysis, it has been proposed that the glycolytic activity of RB is mediated via joint action with MYC [44], and the exact mechanism of action remains to be investigated.

#### Transcription factors with environment-dependent functions

##### TP53

TP53, which is an oncogene, encodes the protein p53, one of the most widely studied transcription factors. It has been reported that the R175H, R248Q and R273 mutations in p53 promote tumour cell glycolysis more than the wild-type protein does, and this phenomenon is associated with elevated protein levels of GLUT1, GLUT3, HK1 and HK2 proteins [45, 46]. In terms of energy metabolism, the regulatory function of p53 depends mainly on the intracellular oxygen concentration. Clinical studies on tumours have shown that severe hypoxia (O2 = 0.02–0.1%) can activate p53 via stress-induced covalent modification, promoting its nuclear accumulation and activating the transcription of downstream glycolysis-related genes [47]. Overexpression of p53 in Saos-2 cells, HeLa cells and mouse embryonic fibroblasts (MEFs) under normoxic conditions (21% O2) results in a significant decrease in the expression of GLUT1, GLUT3 and GLUT4, along with a more than threefold increase in the expression of mitochondrial proteins (2OGDH, GA and ND1). Conversely, unlike normoxic conditions, p53 overexpression under conditions of decreased oxygen concentrations (0.1–1%) leads to increased levels of glycolytic proteins (GLUT1 and GLUT3) [46]. Thus, under conditions of severe hypoxia, p53 mainly facilitates tumour cell glycolysis.

##### KLF14

KLF14, a Krüppel-like transcription factor, is of crucial significance in diverse biological regulatory functions and is involved in multiple pathological mechanisms. Numerous studies have demonstrated that KLF14 plays a pivotal role in lipid metabolism, glucose homoeostasis, and insulin sensitivity. In the context of sepsis, KLF14 modulates the immune function of macrophages by suppressing the transcription of HK2, thereby diminishing glycolysis in macrophages and the secretion of inflammatory cytokines [48]. Moreover, the upregulation of KLF14 has been shown to impede cancer progression, suggesting an inhibitory effect of KLF14 on tumour glycolysis [49]. Through in-depth investigations into the specific mechanisms of KLF14 in various tumour types, the precision and efficacy of tumour treatment could be further enhanced.

### Interactions between transcription factors

**Fig. 3:**
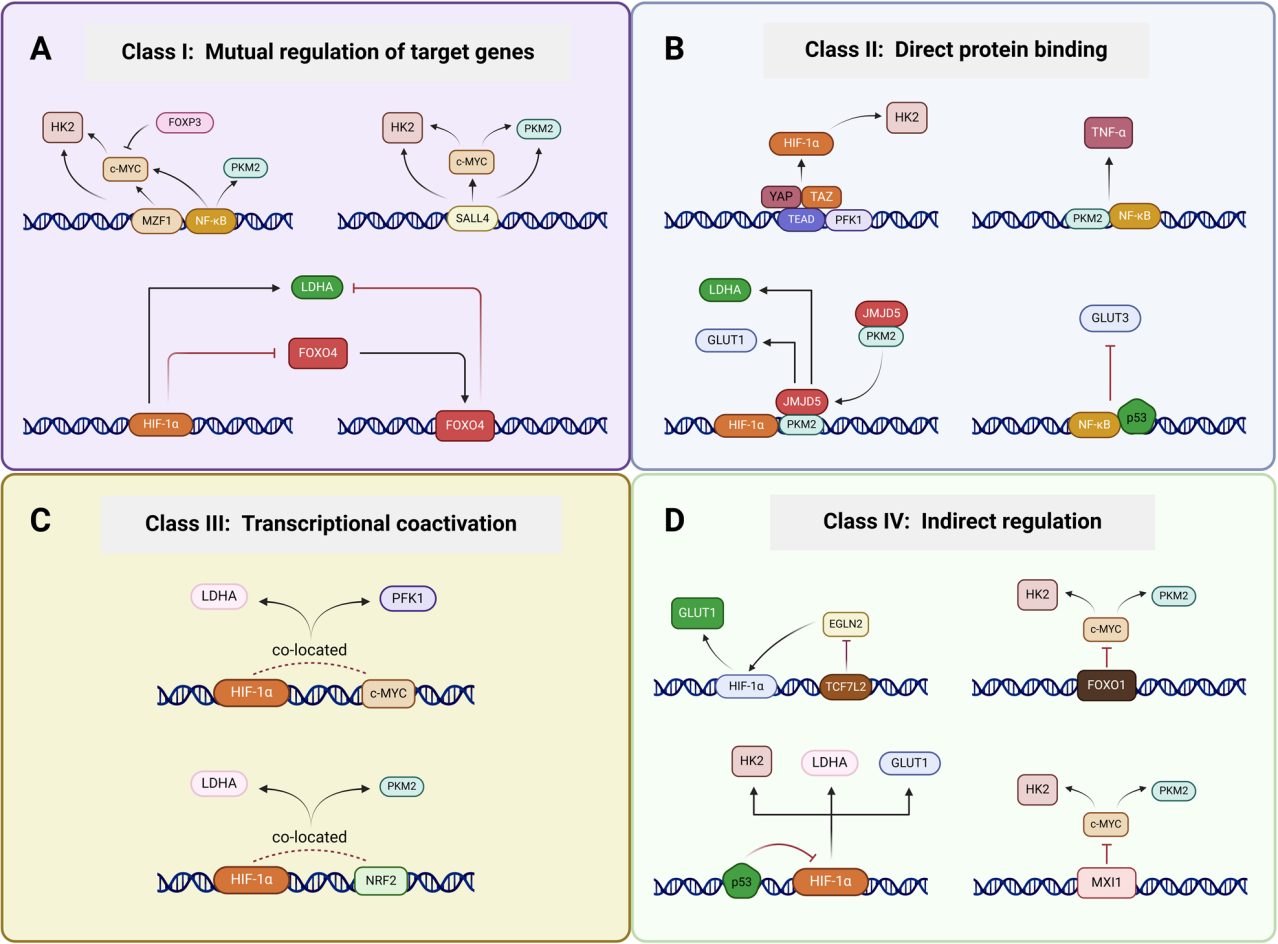
Interactions between transcription factors. **A** Mutual regulation of target genes: MZF1 can directly target HK2 to promote glycolysis and upregulate MYC to achieve the same goal. NF-κB has a similar targeting effect on MYC. Foxp3 inhibits MYC activity and further inhibits glycolysis, but the exact mechanism is unclear. **B** Direct protein binding: The transcription factor TEAD itself is not transcriptionally active and needs to bind to the transcriptional coactivator YAP/TAZ to target HIF-1α for action. NF-κB and monomeric PKM2 form protein complexes and translocate to the nucleus to function as transcription factors. **C** Transcriptional coactivation: NRF2 is often co-expressed with HIF-1α to coactivate the transcription of key enzymes, such as PKM2 and LDHA. **D** Indirect regulation: TCF7L2 positively regulates aerobic glycolysis by mediating the upregulation of HIF-1α through the inhibition of EGLN2 expression. YAP Yes-Associated Protein, TAZ transcriptional co-activator with PDZ-binding motif, TCF7L2 transcription factor-7-like-2, NRF2 nuclear factor erythroid 2-related factor 2. This figure is created with BioRender.com.

As the study of transcription factors that are associated with tumour cell glycolysis has gradually advanced, various transcription factors have been shown to form a complex regulatory network, either synergistically promoting or functionally inhibiting each other, further elucidating the transcriptional regulatory system of the glycolytic pathway. Regulatory interactions between transcription factors include the regulation of other transcription factors via classical functional binding to the promoter regions of downstream genes, indirect regulatory effects via other signalling pathways, interactions as transcription factor complexes, or coactivation, and these interactions depend on the cancer type and experimental model (Fig. 3).

#### Mutual regulation of target genes

Classical transcription factors, such as HIF-1α and MYC, can directly initiate the transcriptional translation of key glycolytic enzymes, such as GLUT1 and HK2, but some transcription factors can also directly upregulate the expression of MYC to promote tumour development. For example, SALL4 transcriptionally activates the MYC oncogene in endometrial cancer cells, further increasing the intracellular levels of the key glycolytic enzymes HK2 and PKM2 and promoting tumour growth and development [50]. Recent studies have shown that MZF1 promotes the transcription of MYC and promotes the growth, migration and invasion of lung adenocarcinoma cells [51]. NF-κB also has a similar regulatory effect on MYC [50]. In endothelial cells (ECs), FOXO1 targets and antagonizes signalling downstream of MYC genes and participates in the regulation of cellular metabolism and cycle processes [52].

Although the direct effects of some transcription factors on tumour cell glycolysis have not been reported, they may still perform protumour functions via other pathways. For example, Foxp3 transcriptionally represses the expression of MYC, which in turn inhibits glycolysis in Treg cells while enhancing OXPHOS and nicotinamide adenine dinucleotide oxidation, allowing Treg cells to adapt to inflammatory environments with low levels of glucose and high levels of lactate [53]. In addition, HIF-1α can directly downregulate FOXO4 expression in gastric cancer (GC) cells, further targeting LDHA to indirectly promote tumour cell glycolysis [54]. In conclusion, directly regulating the transcription of other genes by targeting gene promoter sequences is the primary function of transcription factors, but feedback regulatory mechanisms also occur between individual transcription factors.

#### Direct protein binding

Protein-protein interaction (PPI) refers to the formation of protein complexes between two or more protein molecules through noncovalent bonds, and PPI plays a central role in a variety of biological reactions with widely varying effects. Transcription factors can function alone or in combination with other transcriptional coactivators, forming protein complexes that bind together in the promoter regions of specific genes to initiate transcription.

TEAD transcription factor family members are the ultimate nuclear effector elements of the Hippo pathway; the members of this family are highly conserved in evolution and regulate cell growth, proliferation and drug resistance by regulating the transcription of target genes. Unlike other transcription factors, most TEADs are localized to chromatin, but they exhibit little transcriptional activity; rather, they rely on their C-terminus to recruit the transcriptional coactivators YAP/TAZ to co-initiate the transcription of target genes [55]. Most previous studies suggest that TEAD relies on YAP/TAZ synergy and that the Hippo signalling pathway is involved in the regulation of cellular metabolism, including the promotion of glycolysis, lipogenesis and glutamine catabolism. It has been reported that the removal of glucose from the medium increases the proportion of YAP/TAZ molecules that are phosphorylated and localized to the cytoplasm, thereby inhibiting the nuclear transcriptional process of YAP/TAZ/TEAD, and this may be related to the binding of the PFK1 and TEAD proteins [56]. Additionally, TEADs coregulate the expression of HIF-1α with YAP/TAZ and promote tumour cell glycolysis [57]. Moreover, the glycolytic rate-limiting enzyme HK2, which is a direct downstream target of TEAD, induces breast cancer cell migration by promoting glycolysis [58], and TEAD-binding sites are also present in the promoter regions of MYC and GLUT1 in human leukaemia cells; however, no experimental confirmation is available, and more mechanisms of action are still being explored.

In addition to binding as transcription factor complexes, crosstalk between transcription factors at the protein level affects their function. For example, NF-κB forms a protein complex with monomeric PKM2, stabilizing the interaction between the two proteins and facilitating their nuclear translocation. Similarly, the dioxygenase JumonjiC (JmjC) domain-containing protein 5 (JMJD5) forms a protein complex with monomeric PKM2, inhibits its glycolytic enzyme activity and further translocates to the nucleus; in the nucleus, this complex recruits HIF-1α to form a transcription initiation complex, which induces the transcription of downstream GLUT1, LDHA and other key enzymes to increase glycolysis in breast cancer cells [59]. In addition, NF-κB has been shown to interact with p53 at the protein level. In acute lymphoid leukaemia cells, IkB interacts with p53 to inhibit tumour cell apoptosis via the NF-κB signalling pathway. RelA, which is a member of the NF-κB family, promotes cancer cell glycolysis by upregulating GLUT3 in the absence of p53 [60].

In summary, direct interactions between transcription factors at the protein level often results in functions such as intracellular localization, enzymatic activity and transcriptional translation of DNA.

#### Transcriptional coactivation

Interactions at the protein level include not only direct binding but also synergistic activation of target gene transcription, which means that the binding site in the target gene promoter remains available, playing a “tandem” role. The MYC gene is one of the oncogenes that has been studied for the longest time, and it is upregulated in a variety of cancer cells, such as liver, lung, stomach, breast and colon cancer cells; in these cells, this gene regulates a variety of biological processes, such as cell proliferation, cell cycle progression and apoptosis. Among MYC gene family members, the relevance of MYC to tumour cell glycolysis is stronger and more well studied. At the metabolic level, MYC directly upregulates the expression of the glycolysis-related genes HK2, PFK1, TPI, GAPDH, ENO, LDHA and MCT1, leading to a significant increase in glycolysis in tumour cells [61]. Notably, under hypoxic conditions, MYC tends to act synergistically with HIF-1α to coregulate the expression of downstream target genes [14]. In addition, N-MYC can regulate glycolysis in tumour cells by regulating the expression of the downstream gene N-MYC downstream-regulated gene 2 (NDRG2) to indirectly promote MYC activity [62] or by interacting with HIF-1α to directly upregulate the expression of PGK1, HK2 and LDHA [63]. The transcription factor nuclear factor erythroid 2-related factor 2 (NRF2) is often co-expressed with HIF-1α in breast cancer, and its expression is positively correlated with that of key enzymes such as PKM2 and LDHA.

#### Indirect regulation

In addition to direct effects at the DNA and protein levels, several types of indirect links exist between transcription factors. For example, the presence of FOXO1 in vascular endothelial cells (ECs) inhibits signalling downstream of MYC and impairs glycolysis, OXPHOS and EC proliferation, but the exact mechanism of action is unclear. The oncogene Max-interacting protein 1 (MXI1) is a known MYC transcriptional repressor, and MYC also promotes U87 glioma development by repressing MXI1 via miR-155 and miR-23a in turn [64]. It has been suggested that the transcription factor Transcription factor-7-like-2 (TCF7L2) can positively regulate aerobic glycolysis by suppressing the expression of the Egl-9 family hypoxia-inducible factor 2 (EGLN2) and mediating the upregulation of HIF-1α [65]. There is also an interaction between p53 and HIF-1α, and silencing of p53 results in the loss of the metabolic function of HIF-1α. In conclusion, the complex and diverse modes of action among transcription factors should to be explored and elucidated by more researchers.

## Prospects of glycolysis-related transcription factors in clinical tumour treatment

Glycolysis represents one of the prominent characteristics of metabolic reprogramming in tumour cells. Abundant research findings have indicated that glycolysis occupies a pivotal position in the oncogenesis, progression, and treatment of tumours. Transcription factors associated with glycolysis exert a significant influence in modulating the metabolic pathways of tumour cells, thereby emerging as prospective therapeutic targets. Nevertheless, the employment of glycolysis-related transcription factors for clinical tumour treatment is confronted with a series of challenges and constraints.

To commence with, the elevated expression levels of glycolysis-related transcription factors within tumour cells are frequently correlated with enhanced tumour invasiveness and unfavourable prognoses. For instance, prior investigations have demonstrated that the transcription factor ZEB1, in synergy with the NuRD complex, facilitates glycolysis in colorectal cancer, thereby fuelling tumourigenesis and development [66]. Moreover, glycolysis-related long non-coding RNAs (lncRNAs) also play an indispensable role in tumour energy metabolism and the immune microenvironment, proffering novel therapeutic avenues [67]. Subsequently, the significance of glycolysis within the tumour microenvironment cannot be understated. Glycolysis not only serves as an energy source for tumour cells but also contributes to tumour immune evasion by influencing the infiltration and functionality of immune cells in the tumour microenvironment [68]. Illustratively, in head and neck squamous cell carcinoma, the upregulated glycolysis is intimately linked to immune escape and tumour progression [69]. Nonetheless, treatment strategies centred around glycolysis-related transcription factors are also fraught with challenges. Although glycolysis inhibitors have manifested promising anti-tumour efficacies in certain scenarios, their clinical applications may be circumscribed by tumour heterogeneity and drug resistance [70]. Additionally, the suppression of glycolysis might impinge upon the metabolic functions of normal cells, precipitating potential adverse effects.

In summation, glycolysis-related transcription factors hold substantial potential in the realm of tumour treatment. However, their clinical translation still necessitates further in-depth research and optimization. By comprehensively deciphering the specific mechanisms underlying glycolysis in tumours and integrating other treatment modalities, such as immunotherapy and chemotherapy, the therapeutic outcomes could potentially be enhanced [71]. Concurrently, the application of nanotechnology also unfolds new vistas for regulating tumour glycolysis-related metabolism [72].

## Conclusions and perspectives

In recent years, as research on the regulation of tumour metabolism has progressively expanded, the crucial role of transcription factors within the glycolysis pathway has received escalating attention. The mechanisms of action of classical transcription factors have been intensively investigated, and their target spectra are relatively extensive, encompassing targets at nearly all stages of the glycolysis pathway. However, for certain emerging transcription factors, such as SALL4 and SMAD4, due to the relatively brief research history and a paucity of pertinent reports, a limited number of targets have been identified, thereby necessitating further exploration. These transcription factors possess relatively singular targets and predominantly rely on classical transcription factors to exert their functions. Notably, these transcription factors are capable of modulating tumour cell glycolysis through transcriptional activation of target genes, protein-protein interactions, transcriptional coactivation, or other molecular bridging mechanisms. Moreover, some transcription factors can also establish direct or indirect associations with these transcription factors, and additional interaction modalities may exist; hence, further in-depth research in this domain is warranted.

Enhanced glycolysis constitutes a significant characteristic that differentiates tumour tissues from normal tissues. There are numerous preclinical studies on drugs targeting glycolysis; nevertheless, only a very small fraction of drugs has ultimately advanced to clinical application or reached the market. The majority of these drugs exhibit high toxicity, low specificity, and an unclear mechanism of action. Transcription factors, being a class of proteins with complex and systemic target profiles, confer certain advantages when targeting glycolysis-related transcription factors. Overall, the research vistas of glycolysis-related transcription factors in tumour cells are highly extensive, and the development of more secure and efficacious targeted drugs remains an imperative.
